# Comparison of weight bearing functional exercise and non-weight bearing quadriceps strengthening exercise on pain and function for people with knee osteoarthritis and obesity: protocol for the TARGET randomised controlled trial

**DOI:** 10.1186/s12891-019-2662-5

**Published:** 2019-06-18

**Authors:** Kim L. Bennell, Rachel K. Nelligan, Alexander J. Kimp, Tim V. Wrigley, Ben Metcalf, Jessica Kasza, Paul W. Hodges, Rana S. Hinman

**Affiliations:** 1 Centre for Health, Exercise and Sports Medicine, Department of Physiotherapy, School of Health Sciences, The University of Melbourne, Melbourne, VIC Australia; 2 School of Public Health and Preventive Medicine, Monash University, Melbourne, VIC Australia; 3 Centre for Clinical Research Excellence in Spinal Pain, Injury and Health, School of Health and Rehabilitation Sciences, The University of Queensland, Brisbane, QLD Australia

**Keywords:** Osteoarthritis, Knee, Physiotherapy, Exercise, Obesity, Clinical trial, RCT

## Abstract

**Background:**

Clinical guidelines recommend exercise as a core treatment for individuals with knee osteoarthritis (OA). However, the best type of exercise for clinical benefits is not clear, particularly in different OA subgroups. Obesity is a common co-morbidity in people with knee OA. There is some evidence suggesting that non-weight bearing exercise may be more effective than weight bearing exercise in patients with medial knee OA and obesity.

**Methods:**

To compare the efficacy of two different exercise programs (weight bearing functional exercise and non-weight bearing quadriceps strengthening) on pain and physical function for people ≥50 years with painful medial knee OA and obesity (body mass index ≥30 kg/m^2^) 128 people in Melbourne, Australia will be recruited for a two group parallel-design, assessor- and participant-blinded randomised controlled trial. Participants will be randomly allocated to undertake a program of either weight bearing functional exercise or non-weight bearing quadriceps strengthening exercise. Both groups will attend five individual sessions with a physiotherapist who will teach, monitor and progress the exercise program. Participants will be asked to perform the exercises at home four times per week for 12 weeks. Outcomes will be measured at baseline and 12 weeks. Primary outcomes are self-reported knee pain and physical function. Secondary outcomes include other measures of knee pain, physical function, quality-of-life, participant-perceived global change, physical performance, and lower limb muscle strength.

**Discussion:**

This study will compare the efficacy of two different 12-week physiotherapist-prescribed, home-based exercise programs for people with medial knee OA and obesity. Findings will provide valuable information to help inform exercise prescription in this common OA patient subgroup.

**Trial registration:**

Australian New Zealand Clinical Trials Registry reference: ACTRN12617001013358, 14/7/2017

**Electronic supplementary material:**

The online version of this article (10.1186/s12891-019-2662-5) contains supplementary material, which is available to authorized users.

## Background

Knee osteoarthritis (OA), predominantly affecting the medial tibiofemoral compartment, is a major public health problem [1, 2]. Pain is a dominant characteristic, becoming persistent and more limiting as the disease progresses, resulting in reduced physical function and quality-of-life and often, costly joint replacement surgery [3]. Obesity is a common co-morbidity in individuals with knee OA and is an established risk factor for disease progression [4–8]. With the significant personal, social and economic burden of OA well documented [3, 9], targeted effective conservative treatments for people at high risk of disease progression, such as those with concomitant obesity, are needed to reduce this burden.

Exercise therapy relieves knee pain at all stages of OA [10]. Although similar in magnitude to common drug treatments, effect sizes for exercise are modest [10]. This may be because randomised controlled trials (RCT) have generally used a “one-size-fits-all” approach to exercise prescription, and have not tailored exercise according to clinical presentation, resulting in attenuation of treatment effects. Our previous study [11] provides preliminary evidence supporting this premise. We compared two different types of exercise programs, weight bearing neuromuscular and non-weight bearing quadriceps strengthening, in individuals with medial knee OA. Modest improvements in pain with both programs were not significantly different between the two. However, exploratory post-hoc analyses showed that people with obesity (body mass index ≥30 kg/m^2^) had different pain relieving responses from the two exercise programs [12]. Specifically, a greater benefit for pain was found with a non-weight bearing quadriceps exercise program than with a weight bearing program. These hypothesis-generating findings suggest that individuals with medial knee OA and obesity may require specific types of exercise in order to maximise outcomes.

The mechanism(s) underpinning a potential greater benefit from non-weight bearing exercise than with weight bearing exercise in this OA patient subgroup is not clear. One possible biomechanical mechanism may be attributed to the greater load placed on the knee joint in individuals with a higher body mass index [13]. During weight bearing activities such as walking, climbing stairs, getting in and out of a chair forces through the knee can be 2–3 times body weight [14]. Therefore, the higher a person’s weight the greater the load placed on the knee joints during weight bearing activities. High rates of pain catastrophising, fear avoidance and kinesiophobia in obese individuals has also been documented and may provide another plausible link between higher reported musculoskeletal symptom severity and obesity [15, 16]. We speculate that individuals with painful knee OA and obesity might respond better in terms of pain and function when completing a non-weight bearing (lower knee load), less psychologically threatening form of exercise for their knee OA than a more complex and physically demanding weight bearing program.

The primary aim of this RCT is to directly compare the effects of two different home-based exercise programs, non-weight bearing quadriceps strengthening and weight bearing functional exercise on pain and physical function for individuals with medial compartment knee OA and obesity. We hypothesise that participants who undertake non-weight bearing quadriceps strengthening will have greater improvements in pain and function than those who undertake weight bearing exercise at 12 weeks. Our secondary aim is to compare the effectiveness of the two different exercise programs on a range of other outcomes such as other measures of pain, function, health-related quality-of-life, global change, muscle strength and physical performance.

## Methods/design

### Trial design

**Fig. 1:**
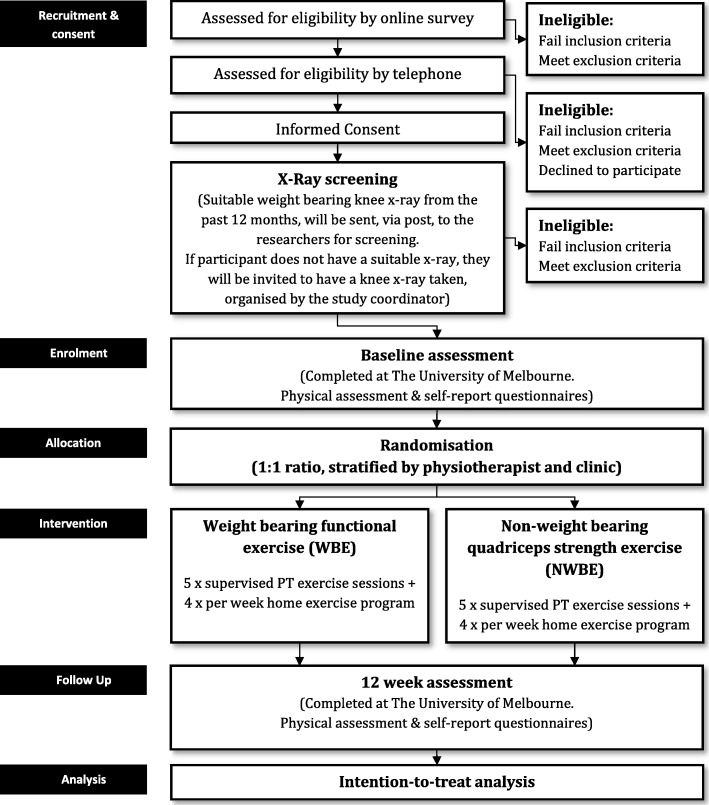
Participant flow through the randomised controlled trial

This protocol is described according to SPIRIT guidelines for clinical trials [17]. The TARGET trial is a two-arm parallel-groups design, assessor- and participant-blinded RCT. Reporting of the trial will comply to CONSORT [18, 19] and TIDieR [20] guidelines. Fig. 1 outlines the RCT phases. The trial will be conducted at The University of Melbourne over 2 years. Assessments will be performed at baseline and 12 weeks.

### Participants

A total of 128 participants aged ≥50 years with painful medial knee OA and obesity (body mass index (BMI) ≥ 30 kg/m^2^) will be recruited from the community in Melbourne, Australia. Potential participants will be identified by: (1) mail out or emails sent to research volunteers on existing databases who have consented to be contacted, (2) flyers or posters placed on the notice boards of local clubs, on waiting room walls in medical, radiology or private physiotherapy clinics, (3) paid advertisements or free postings on Facebook, (4) local and major newspaper advertisements, and (5) radio or television interviews with investigators. Participants will be included if they:

i) Are aged ≥50 years;
ii) Report knee pain on most days of the past month;
iii) Have had knee pain for 3 months or more;
iv) Report a minimum average overall pain severity of 4 on an 11-point numeric rating scale (NRS) over the previous week;
v) Demonstrate tibiofemoral osteophytes on x-ray;
vi) Are obese (BMI ≥ 30 kg/m^2^);
vii) Have a mobile phone that has text messaging functionality and are happy to receive text message reminders if required during the study.

Exclusion criteria will include:

i) Lateral joint space narrowing greater than or equal to medial joint space narrowing on x-ray according to a radiographic atlas [21] (where Grade 0 = no narrowing, 1 = mild narrowing, 2 = moderate narrowing, 3 = severe narrowing);
ii) Knee surgery/joint injection in past 6 months or planned surgery in the next 9 months;
iii) Current or past (4 weeks) oral corticosteroids use;
iv) Systemic arthritic conditions;
v) Past knee fracture or malignancy;
vi) Past hip/knee joint replacement/tibial osteotomy;
vii) Other condition currently affecting lower limb function;
viii) Participation in knee strengthening or neuromuscular/functional exercise in past 6 months or planning to start exercise in next 9 months;
ix) Unable to walk unaided;
x) Unable to commit to study requirements.

### Procedures

Volunteers will undergo screening via an online survey, followed by telephone screening, to ensure eligibility. Potentially suitable volunteers will then be invited to undergo radiographic screening. If participants have a suitable weight bearing knee x-ray from the past 12 months, this can be used for screening. If participants do not have a suitable recent x-ray, they will be invited to have a new knee x-ray taken.

Baseline and follow-up assessments (completed at the Department of Physiotherapy in the University of Melbourne) will be conducted by the same assessor blinded to exercise group allocation. Participants will visit a physiotherapist five times over the 12-week intervention. Only one knee will be the focus of treatment and evaluation. For participants with bilateral symptoms, the most symptomatic eligible knee, will be nominated. In cases where bilateral knees are equally symptomatic, the right knee will be nominated. Participants will be advised to continue with their usual medication during the trial. Regular study newsletters and Christmas cards will be sent to help with participant retention. Ethical approval has been obtained from the Human Research Ethics Committee of the University of Melbourne (HREC No. 1544919) and all participants will provide written informed consent.

### Data collection and management

Data will be obtained via online questionnaires (or paper-based if requested). Data will be stored in secure electronic databases and de-identified. Some data (i.e. Physiotherapists’ treatment notes) will be stored either in hard copy or electronically on the premises of the treating physiotherapist. Offsite storage will be in accordance with the protocols for maintaining security and privacy of data and will be password protected. Once completed, the notes will be mailed/emailed to researchers to be stored securely. All authors will have access to the final trial dataset.

### Randomisation allocation concealment and blinding

Randomisation will occur according to a 1: 1 allocation ratio and be stratified by combination of study physiotherapist and their physiotherapy clinic (two physiotherapists worked at two separate clinics each). On completion of baseline assessment at The University of Melbourne, participants will be randomly allocated into one of the two interventions groups: i) non-weight bearing quadriceps strengthening exercise program (NWBE); or ii) weight bearing functional exercise program (WBE).

The randomisation schedule will be computer generated, using random permuted blocks of sizes 6 to 12, by a person not involved in recruitment of participants. The randomisation schedule will only be accessed via a password-protected computer program. The person who will determine if a potential participant is eligible for inclusion in the trial will be unaware, when this decision is made, as to which group the participant will be allocated.

Participants will be blinded to study hypotheses and informed that we are comparing two different exercise programs for people with knee OA. We will not disclose details of either exercise program prior to randomisation. After randomisation, participants will only be provided with details of the exercise program they will be undertaking.

The person administering physical function outcomes assessment will be blinded. As questionnaire-based outcomes are self-reported, and participants are blinded, this study is also considered assessor blinded. The person performing the statistical analyses will be blinded.

Group allocation can be immediately unblinded if deemed necessary by the Chief Investigator in the case of any unexpected adverse events related to the study.

### Interventions

Seven physiotherapists in seven private practices at various locations throughout Melbourne, Australia will deliver both interventions. The physiotherapists have an average of 15.1 (range 6–28) years of clinical experience since qualification and 10.6 (range 3–23) years of post-graduate clinical musculoskeletal experience. Six (85%) have formal postgraduate Masters qualifications in sports or manipulative/musculoskeletal therapy. All physiotherapists will attend a 4-h training session and be provided with a treatment manual describing the interventions. Regular telephone meetings will be conducted with the physiotherapists to reinforce the protocol and discuss any study issues. This will help to ensure similar treatment administration among the therapists. Weights and elastic bands will be provided to the physiotherapist to give to the study participants.

Participants in both groups will visit a physiotherapist for an individual session five times over 12 weeks (approximately study weeks 1, 2, 4, 7 and 10). Each session will last 30–40 min. In our previous study, participants visited the physiotherapist on 14 occasions over 12 weeks [11]. However we reduced this to five sessions in the current study for several reasons. First, informal feedback from participants was that the number of sessions was excessive. Second, our other exercise studies in patients with knee OA have shown comparable adherence with fewer sessions [22, 23]. Third, five sessions is a more realistic number to generalise to real life clinical practice. The physiotherapist will teach the participant an exercise program which they will be asked to perform at home four times per week. At the scheduled sessions, the physiotherapist will conduct a brief reassessment to determine progress and any adverse effects and will watch the participant complete the program in order to review and correct quality and form of exercise performance. Findings will help determine physiotherapists’ decision-making regarding progression or modification of the exercises. Participants will be asked to record their exercise completions in a log book. The physiotherapists will check the participants’ log book and set goals/targets to help maintain adherence and motivation. Both groups will also receive a one-page sheet providing information about OA.

#### Non-weight bearing quadriceps strengthening exercise program (NWBE)

The aim of the non-weight bearing quadriceps strengthening exercise program (NWBE) is to improve the strength of the quadriceps. The program consists of exercises performed in sitting or supine where the participant is not bearing their body weight through the affected lower limb (Additional file 1) and is based on those used in our previous RCT’s [11, 24, 25].

Participants will be provided with adjustable ankle cuff weights (0.65 kg to 10 kg) and elastic resistance bands. They will commence the program with two sets of ten repetitions for each exercise for the first 2 weeks and progress to three sets thereafter or as quickly as able. The starting weight will be the participant’s 10-repetition maximum weight or determined by the participant’s level of effort aiming for between 5 and 8 out of 10 (hard to very hard) on the modified Borg Rating of Perceived Exertion CR-10 scale for strength training [26]. Participants will be instructed that each exercise should be performed slowly in a controlled manner. Progression will be guided by the physiotherapist at regular intervals with adjustments to participants’ ankle weights or elastic resistance bands. Participants will also be encouraged to increase the weights (0.5 kg at a time) for each exercise if they feel the exercise is ‘easier to complete’ compared to the beginning of that week. The end position of each exercise is to be held initially for 5 s and then increased to 10 s.

Participants will be advised that the exercises should be performed within a tolerable level of pain. Some discomfort is expected, but by the next day pain should subside to usual levels with no increase in knee swelling following the exercise session. If the physiotherapist feels that a particular exercise is exacerbating the participant’s pain, then the physiotherapist may reduce the resistance, dosage and/or level of exercise until the pain aggravation settles. If joint swelling or increased pain lasting more than 1 day occurs, the program should be modified by reducing the intensity, frequency and/or number of repetitions by half. If pain or swelling is excessive and the therapist deems it appropriate, the exercise program can be ceased for a period of time.

#### Weight bearing functional exercise program (WBE)

This program incorporates neuromuscular exercises. The aim is to functionally strengthen the lower limb muscles, improve trunk/lower limb joint alignment and quality of movement performance. The exercises are based on those used in our previous RCT [11, 25]. The five exercises and their progressions are described in detail in Additional file 1. Participants will be provided with an adjustable step (10 cm to 15 cm height), foam mat, and elastic resistance bands.

A major emphasis is on the quality of performance of each exercise. Participants will be instructed to maintain alignment of the pelvis and trunk in the frontal plane (avoid pelvic drop and trunk lateral flexion) and to neutrally align their knee (position the knee over the foot as much as possible) throughout the movements. Knee flexion should not exceed 30° (except when performing the chair stand exercise) to reduce the risk of increasing knee pain. Having hand support within easy reach or using hand support is important for maintaining balance and quality of performance throughout the movements. Exercise progression is essential and is determined by the physiotherapist, based on a combination of the participant’s pain and rating of perceived exertion score for each exercise (at least 5 out of 10 on a modified Borg Rating of Perceived Exertion CR-10 scale [26]) and the physiotherapist’s assessment of the quality of the exercise performance. Exercises should be performed within a tolerable level of pain as per the method outlined for the non-weight bearing exercise program. Progressions will occur by increasing resistance, changing stance surface, and/or varying the repetitions, direction, and speed of movements. Some participants may not reach the final level of each exercise depending on their rate of progression.

### Measures

Table 1 outlines the outcomes being measured, and the time-points for measurement. The primary outcomes are person-centred, reliable and valid measures recommended for knee OA trials [27, 28].

**Table 1:**
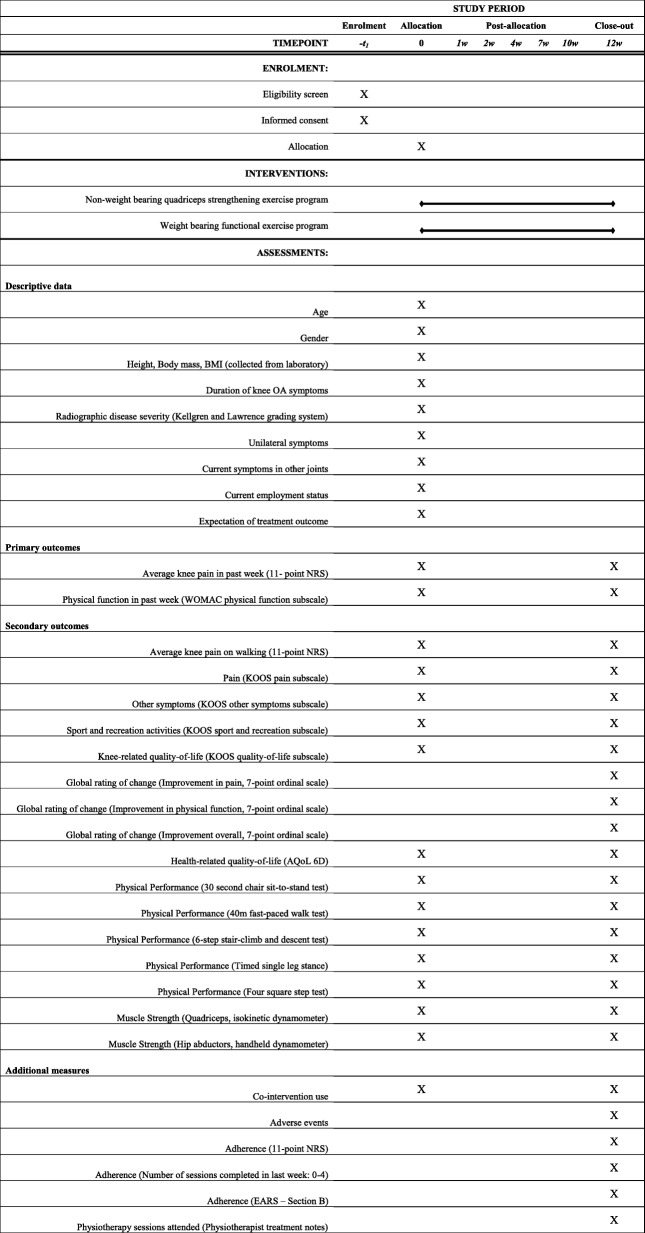
Schedule of enrolment, interventions, and assessments

*BMI* Body Mass Index, *NRS* numeric rating scale, *WOMAC* Western Ontario and McMaster Universities Osteoarthritis Index, *KOOS* Knee Injury and Osteoarthritis Outcome Score, *AQoL 6D* Assessment of Quality of Life Instrument, *EARS* Exercise Adherence Rating Scale

#### Descriptive data

Age, gender, duration of knee OA symptoms, previous treatments, current symptoms in other joints, medical history, medication use, current employment status and expectation of treatment outcome will be obtained at baseline by questionnaire. Measures of height and body mass will also be collected in the laboratory and body mass index will be calculated. Radiographic disease severity will be assessed using the Kellgren and Lawrence grading system [29].

#### Primary outcomes

##### Pain

Overall average pain severity in the past week will be measured via an 11-point NRS with terminal descriptors of ‘no pain’ (score 0) and ‘worst pain possible’ (score 10) [30].

##### Physical function

The Western Ontario and McMaster Universities (WOMAC) Osteoarthritis Index (Likert version 3.1) [31] will be used to measure limitations with physical functioning over the past week. This is a self-report, disease-specific instrument with established validity, reliability and responsiveness [32]. The physical function subscale contains 17 questions with Likert response options ranging from 0 (none) to 4 (extreme). WOMAC scores will be extracted from the Knee Injury and Osteoarthritis Outcome Score (KOOS) [33] questionnaire, which contains the WOMAC questions. Total scores range from 0 to 68, with higher scores indicating worse function.

#### Secondary outcomes

##### Walking pain

Average knee pain severity on walking in the past week will be measured with an 11-point NRS with terminal descriptors of ‘no pain’ (score 0) and ‘worst pain possible’ (score 10) [30].

##### Pain subscale of the KOOS

This is scored using nine questions about knee pain experienced in the last week, with Likert response options from None to Extreme [33]. Scores range from 0 to 100 with lower scores indicating worse pain.

##### Other symptoms subscale of the KOOS

The subscale is scored using seven questions about knee symptoms experienced in the last week, with Likert response options from None to Extreme [33]. Scores range from 0 to 100 with lower scores indicating worse symptoms.

##### Sport and recreation subscale of the KOOS

This is scored using five questions about difficulty with sport and recreational activities in the last week, with Likert response options ranging from None to Extreme [33]. Scores range from 0 to 100 with lower scores indicating greater difficulty.

##### Knee-related quality-of-life subscale of the KOOS

This is scored using four questions about knee-related quality of life experienced in the last week, with Likert response options ranging from Never to Extreme [33]. Scores range from 0 to 100 with lower scores indicating lower quality of life.

##### Global change

Using 7-point Likert scales, participants will rate their change in pain, change in physical function and change overall since baseline. The terminal descriptors will be ‘much worse’ to ‘much better’ [34]. Participants reporting that they are “moderately better” or “much better” will be classified as “improved”.

##### Health-related quality-of-life

This will be measured using the Assessment of Quality of Life – 6-Dimension (AQoL-6D) [35] which comprises 20 items that assess independent living, mental health, relationships, pain, coping and senses. Scores range from − 0.04 to 1.00 with higher scores indicating better quality-of-life.

#### Physical performance

- *30-s chair sit-to-stand test:* The number of complete chair stands (up and down = one stand) completed in 30 s will be counted [36]. Higher scores indicate greater physical function.
- *40 m fast-paced walk test:* The total time taken to walk 4 × 10 m quickly but safely, excluding turns, will be expressed as speed in m/s [36]. Higher walking speeds indicate greater physical function.
- *6-step stair-climb and descent test:* The total time taken to ascend and descend a flight of 6 stairs as quickly and safely as possible will be measured [37]. Use of one handrail is permitted if required. Shorter times to complete the test indicate greater physical function.
- *Timed single leg stance:* Time able to stand on a single limb is measured (up to 30 s) [38]. The best of two repetitions is recorded. Longer times balancing on the single limb indicate greater balance.
- *Four square step test:* Time taken to step a full circle once in each direction while facing forward around four squares created on the ground by walking sticks will be measured [39]. The average of two repetitions is recorded. Shorter times to complete the test indicate greater physical function.

#### Muscle strength

- *Quadriceps:* Maximum voluntary isometric strength of the knee extensors will be assessed on an isokinetic dynamometer (HUMAC, CSMI, Boston) with the knee at 90 degrees of knee flexion [40]. Maximum torque reached over 3 repetitions of 3 s each will be recorded and normalised to body mass (Nm/kg).
- *Hip abductors:* Maximum voluntary isometric strength of the hip abductors recorded using a handheld dynamometer (Lafayette Manual Muscle Test System, Lafayette, Indiana) with the hip in neutral abduction will be assessed. Average force from 2 repetitions of 3 s each will be recorded, converted to torque and normalised to body mass (Nm/kg) [25, 41].

#### Additional measures

##### Co-intervention use

Medications for knee pain and other treatments for knee OA will be recorded at baseline and 12 weeks. Participants will complete a custom-developed table about the frequency of use (over the past 6 months at baseline and the past 12 weeks at follow up) of a number of pain and arthritis medications and co-interventions.

##### Adverse events

These will be ascertained by questionnaire at 12 weeks and defined as any problem that participants believe was caused by the exercise program that required them to seek treatment and/or lasted for two or more days.

##### Adherence

Adherence to the prescribed home exercise program will be self-reported and measured in three ways: i) using an 11-point NRS scale from “strongly disagree” to “strongly agree”, participants will rate to what extent they agree with three statements (“I have been doing my exercise sessions 4 times each week as recommended”; “Within each exercise session, I have been doing all of the exercises recommended (e.g. 5 different exercises)”; “For each exercise, I have been doing the number of repetitions recommended (e.g. ten times each)”); ii) Number of prescribed exercise sessions completed in the last week measured at 12 weeks with scores ranging from zero to four; iii) Exercise Adherence Rating Scale (EARS) Section B [42]. Scores range from 0 to 42, with higher scores indicating better adherence.

##### Number of physiotherapy sessions attended

This will be collected from the physiotherapist treatment notes.

#### Other measures

A range of other measures will be collected for subsequent analyses of potential moderators and mediators of clinical effects. These measures will not be used to determine treatment efficacy. The measures include: physical activity levels using the Physical Activity Scale for the Elderly [43]; self-efficacy using the Arthritis Self Efficacy Scale [44]; kinesiophobia measured with the Brief Fear of Movement Scale for OA [45]; pain catastrophising using the Pain Catastrophising Scale [46]; emotional state using the Depression, Anxiety, and Stress Scale (DASS-21, [47]); number of knee pain zones assessed using the Photographic Knee Pain Map [48]; pain while performing exercises assessed using an 11-point NRS; knee confidence and knee stability each scored on a 5-point Likert scale.

### Trial sample size

The sample size was calculated based on both primary outcomes of pain and function. For an effect size of 0.5, power 80% and two-sided significance level 0.05, with a correlation between pre- and post-measurements of 0.45 for pain [11], 51 participants per arm will be required (using analysis of covariance including baseline pain measurement as a covariate). To account for 20% loss to follow up, sample size will be increased to 64 per arm, for a total of 128. This gives power of 83% to detect an effect size of 0.5 for function with a correlation between pre- and post-measurements of 0.49 [11] and a two-sided significance level of 0.05.

### Statistical analysis

Analyses comparing the two treatment groups will be performed by the statistician (JK) in a blinded fashion using all available data from all randomised participants. Demographic and baseline characteristics of participants will be summarised as appropriate (means and standard deviations for continuous variables that appear to be approximately normally distributed, medians and interquartile ranges for other continuous variables, counts and percentages for categorical variables) and will be inspected to assess baseline comparability of treatment groups. For continuous outcomes, differences in change will be compared between groups using linear regression models, adjusted for baseline levels of these outcomes, including random effects for treating physiotherapist to account for clustering by physiotherapist. Model assumptions will be assessed using standard diagnostic plots. For binary outcomes, differences between groups will be compared using relative risks, calculated from logistic regression models with random effects for treating physiotherapist [49]. Should the amount of missing data for an outcome be such that imputation is required, multiple imputation will be conducted and the method reported [50].

### Monitoring

Fortnightly meetings will be held between the trial coordinator and the lead investigators to monitor adverse events, and/or any problems that may arise during the course of the trial and to review recruitment targets and timelines. Regular contact between the investigators and the physiotherapists will monitor any problems arising with implementation of the interventions.

### Dissemination plans

Findings of the RCT will be presented at relevant conferences and published in a relevant peer-reviewed journal. Participants with knee OA will be provided with a lay summary of findings. Findings will also be disseminated through the networks of the Centre for Health, Exercise and Sports Medicine, and the National Health and Medical Research Council Centre for Research Excellence in Translational Research in Musculoskeletal Pain.

## Discussion

This trial will provide evidence to show whether there is a difference in clinical outcomes following two different types of exercise programs in people with medial knee OA and concomitant obesity, a common knee OA subgroup. Specifically, it will either prove or disprove the hypothesis that greater benefits will be found with non-weight bearing quadriceps strengthening exercise than with weight bearing functional exercise when applied to this group. The study will help inform exercise prescription recommendations for people with medial knee OA and obesity.

## Additional file

Additional file 1: NWBE and WBE programs. (DOC 749 kb)

## Data Availability

Not applicable.

## Abbreviations

AQoL 6D: Assessment of Quality of Life Instrument
BMI: Body mass index
CONSORT: Consolidated Standards of Reporting Trials
DASS-21: Depression, Anxiety, and Stress Scale
EARS: Exercise Adherence Rating Scale
HREC: Human Research Ethics Committee
KOOS: Knee Injury and Osteoarthritis Outcome Score
NRS: Numeric rating scale
NWBE: Non-weight bearing quadriceps strengthening exercise program
OA: Osteoarthritis
RCT: Randomised controlled trial
TIDieR: Template for Intervention Description and Replication
WBE: Weight bearing functional exercise program
WOMAC: Western Ontario and McMaster Universities Osteoarthritis Index
