# Supplementary material for: Comparison of weight bearing functional exercise and non-weight bearing quadriceps strengthening exercise on pain and function for people with knee osteoarthritis and obesity: protocol for the TARGET randomised controlled trial
Source: BMC Musculoskelet Disord. 2019 Jun 18;20:291. doi: 10.1186/s12891-019-2662-5 (PMC6580522; doi:10.1186/s12891-019-2662-5)
Supplement: Supplementary file 1 — NWBE and WBE programs. (DOC 749 kb) [file 12891_2019_2662_MOESM1_ESM.doc]

**Additional file 1**

Table of contents

|  | Page |
| --- | --- |
| Appendix 1: Non-weight bearing quadriceps strengthening exercise program | 2 |
| Appendix 2: Weight bearing functional exercise program | 7 |
| Appendix 3: Weight bearing functional exercise program progression | 27 |

**Appendix 1 - Non-weightbearing quadriceps strengthening exercise program**

1. Quads over a roll (inner range knee extension)

| Starting position:  Attach weight around ankle of the arthritis leg.  Sit on firm surface with body weight supported by arms behind. You can lie down if you prefer. Bend up the non-study leg and support the knee of the arthritis leg over some rolled up towels. Your arthritis knee should be bent about 30°.  Pull up the foot and ankle on the arthritis leg and keep the knee cap and toes pointing toward the ceiling. | 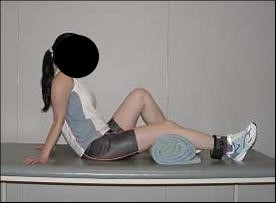  **Arthritis knee** |
| --- | --- |
| Exercise:  Keeping the knee in contact with the towel, straighten the arthritis leg by SLOWLY lifting the heel off the surface.  Hold the leg straight, then SLOWLY lower down. | **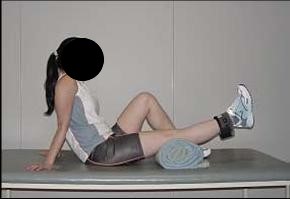** |

2. Knee extension in sitting

| Starting position:  Attach weight around ankle of the arthritis leg.  Sit over the edge of a bed or chair, with both legs off the ground. If your chair is not high enough to have your feet clear of the ground, you can put some phone books on the chair. | 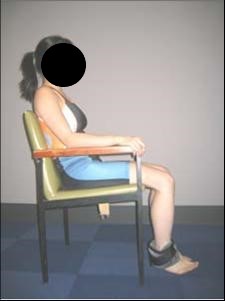  **Arthritis knee** |
| --- | --- |
| Exercise:  SLOWLY straighten the knee and lift ankle weight up as high as possible.  Hold, then SLOWLY lower down. | 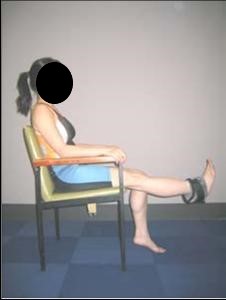 |

3. Knee extension with hold at 30°

| Starting position:  Attach weight around ankle of the arthritis leg.  Sit over the edge of a bed or chair, with both legs off the ground. If your chair is not high enough to have your feet clear of the ground, you can put some phone books on the chair. | 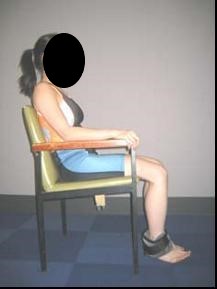  **Arthritis knee** |
| --- | --- |
| Exercise:  Straighten the knee and SLOWLY lift ankle weight up to about 30° short of fully straightening your knee (your knee should be slightly bent).  Hold, then SLOWLY lower down. | 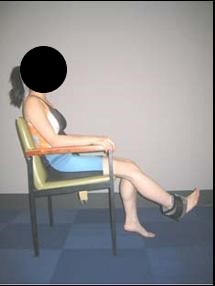  **30°** |

4. Straight Leg Raise

| Starting position:  Attach weight around ankle of the arthritis leg.  Lie on your back with body weight supported by your arms and elbows. You can lie down if you prefer.  Bend up the non-study leg and keep the arthritis leg straight with ankle and foot pulled up towards you. Both the kneecap and toes should be pointing toward the ceiling. | 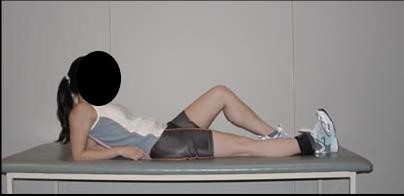  **Arthritis knee** |
| --- | --- |
| Exercise:  SLOWLY raise your leg straight up until it gets to approximately 30 cm or 12 inches off the bed. Ensure you keep your knee straight.  Hold, then SLOWLY lower down. | 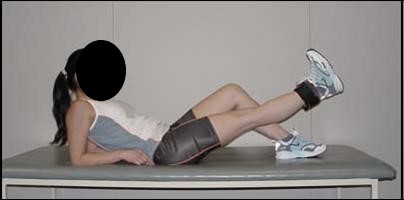 |

**5. Short Arc Knee Extension**

| Starting position:  Your physiotherapist will give you a rubber band tied into a loop. Place the looped rubber band around the leg of a chair.  Sit on the chair and put your leg into the looped rubber band. | 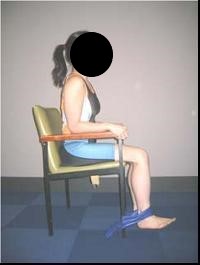  **Arthritis knee** |
| --- | --- |
| Exercise:  Slowly straighten your leg up into the rubber band until you can feel a comfortable resistance (should be about 60° knee bend).  Hold.  Slowly return to the starting position. | 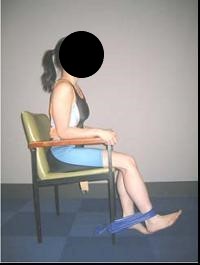 |

**Appendix 2 – Weightbearing functional exercise program**

**Exercise 1: Forwards/backwards exercise**

**Level 1:** Sliding

| Starting position:  Standing on your arthritis leg with the non study leg on a sliding surface.  Sliding can be achieved by using a towel on smooth flooring or a plastic bag on the foot for carpet.  Use hand support for balance. | 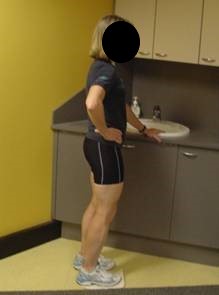 | |
| --- | --- | --- |
| Exercise:  **Slowly** slide backwards and forwards with the ‘sliding leg’ while bending and straightening the arthritis leg.  Start with sliding just a few inches forwards and backwards and progress to larger slides as you gain control.  Keep your weight on the arthritis leg. | 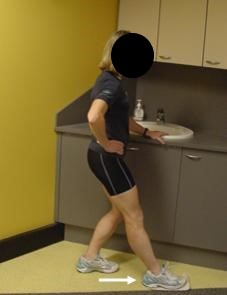 | 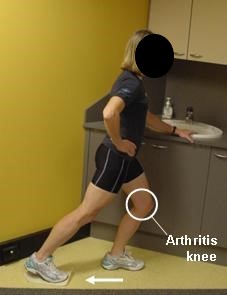 |
| Concentrate on the alignment of your arthritis leg hip, knee and ankle – **position your knee over your foot** throughout.  Do 3 sets of 10 repetitions with a break of 30-60 seconds between sets. | | |

**Exercise 1: Forwards/backwards exercise**

**Level 2:** Sliding with Thera-band

| Starting position:  Place a loop of Thera-Band around your arthritis knee and the leg of a table. This will provide a pull outwards on your knee that you must resist by aligning your knee over your foot through the whole exercise.  Your non study side foot should be on a sliding surface.  Sliding can be achieved by using a towel on smooth flooring or a plastic bag on the foot for carpet. | 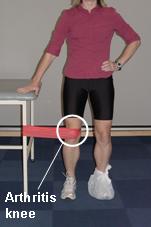 | |
| --- | --- | --- |
| Exercise:  **Slowly** slide backwards and forwards with the ‘sliding leg’ while bending and straightening the arthritis leg.  Start with sliding just a few inches forwards and backwards and progress to larger slides as you gain control.  Keep your weight on the arthritis leg. | 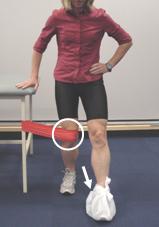 | 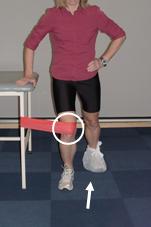 |
| Concentrate on the alignment of your arthritis leg hip, knee and ankle – **position your knee over your foot** against the pull of the Thera-band throughout.  Do 3 sets of 10 repetitions with a break of 30-60 seconds between sets. | | |

**Exercise 1: Forwards/backwards exercise**

**Level 3:** Stepping

| Starting position:  Standing on your arthritis leg with your non-study leg behind.  Use a hand support for balance. | 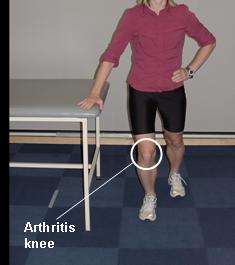 | | |
| --- | --- | --- | --- |
| Exercise:  Instead of sliding forwards and backwards, **slowly** take a step forwards with your non-study leg to touch the floor. Then take a step backwards to the starting position.  Keep your arthritis knee slightly bent and your weight on your arthritis leg through the whole exercise. | 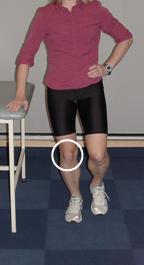 | 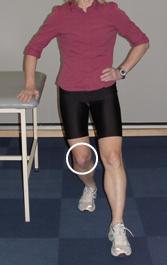 | 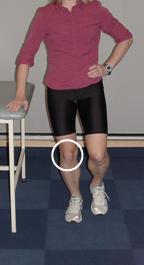 |
| Start with a small step and progress to larger steps as you gain control.  Concentrate on the alignment of your arthritis leg hip, knee and ankle – **position your knee over your foot** throughout.  Do 3 sets of 10 repetitions with a break of 30-60 seconds between sets. | | | |

**Exercise 1: Forwards/backwards exercise**

**Level 4:** Stepping with Thera-band

| Starting position:  Place a loop of Thera-Band around your arthritis knee and the leg of a table. This will provide a pull outwards on your knee that you must resist by aligning your knee over your foot through the whole exercise.  Start standing on your arthritis leg with non-study leg behind.  Use hand support for balance. | | 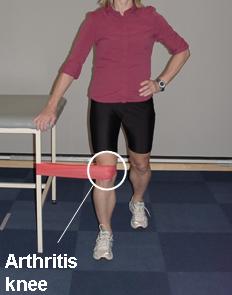 | | |
| --- | --- | --- | --- | --- |
| Exercise:  Slowly, take a step forwards with your non-study leg to touch the floor. Then take a step backwards to the starting position.  Keep your arthritis knee slightly bent and your weight on your arthritis leg through the whole exercise. | 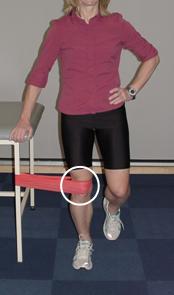 | | 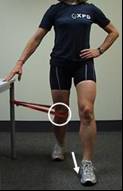 | 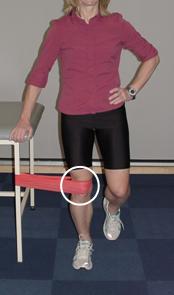 |
| Start with a small step and progress to larger steps as you gain control.  Concentrate on the alignment of your arthritis leg hip, knee and ankle – **position your knee over your foot** against the pull of the Thera-band throughout.  Do 3 sets of 10 repetitions with a break of 30-60 seconds between sets. | | | | |

**Exercise 2: Sideways exercise**

**Level 1:** Sliding

| Starting position:  Standing on your arthritis leg with the other leg on a sliding surface.  Sliding can be achieved by using a towel on smooth flooring or a plastic bag on the foot for carpet.  Use hand support for balance. | 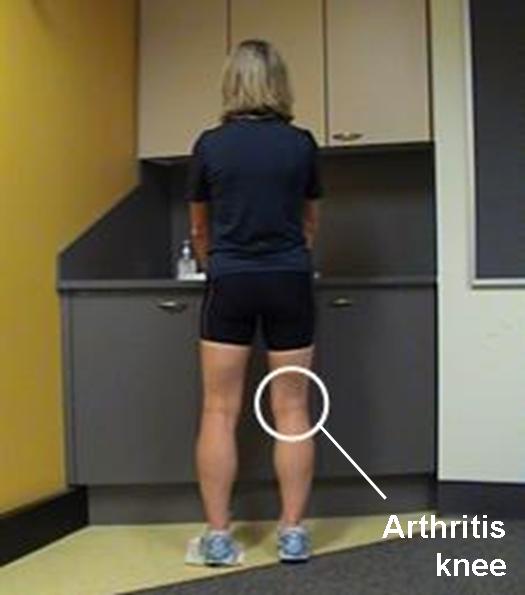 |
| --- | --- |
| Exercise:  **Slowly** slide out sideways with the ‘sliding leg’ while bending the arthritis leg. Then slide back to the starting position.  Start with sliding just a few inches and progress to larger slides as you gain control.  Keep your weight on your arthritis leg through the whole exercise. | 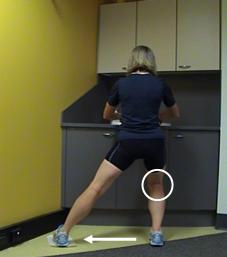 |
| Concentrate on the alignment of your arthritis leg hip, knee and ankle – **position your knee over your foot** throughout.  Do 3 sets of 10 repetitions with a break of 30-60 seconds between sets. | |

**Exercise 2: Sideways exercise**

**Level 2:** Sliding with Thera-band

| Starting position:  Place a loop of Thera-Band around your arthritis knee and the leg of a table. This will provide a pull outwards on your knee that you must resist by aligning your knee over your foot through the whole exercise.  Standing on your arthritis leg with the other leg on a sliding surface.  Sliding can be achieved by using a towel on smooth flooring or a plastic bag on the foot for carpet. | 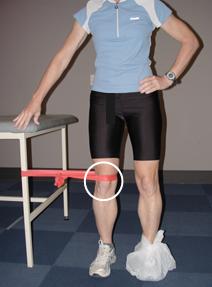  **Arthritis knee** |
| --- | --- |
| Exercise:  **Slowly** slide out sideways with the non-study leg while bending your arthritis knee.  Start with sliding just a few inches and progress to larger slides as you gain control.  Keep your weight on your arthritis leg through the whole exercise. | 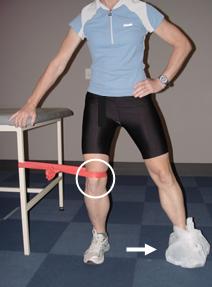 |
| Concentrate on the alignment of your arthritis leg hip, knee and ankle – **position your knee over your foot** against the pull of the Thera-band throughout.  Do 3 sets of 10 repetitions with a break of 30-60 seconds between sets. | |

**Exercise 2: Sideways exercise**

**Level 3:** Sliding with Thera-band and foam

| Starting position:  Place a loop of Thera-Band around your arthritis knee and the leg of a table. This will provide a pull outwards on your knee that you must resist by aligning your knee over your foot through the whole exercise.  Standing on your arthritis leg on your **foam cushion**, with your non-study leg on a sliding surface.  Sliding can be achieved by using a towel on smooth flooring or a plastic bag on the foot for carpet. | 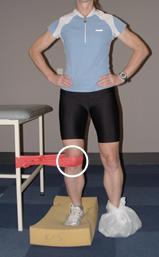  **Arthritis knee** |
| --- | --- |
| Exercise:  **Slowly** slide out sideways with the non-study leg while bending your arthritis knee.  Start with sliding just a few inches and progress to larger slides as you gain control.  Keep your weight on your arthritis leg through the whole exercise. | 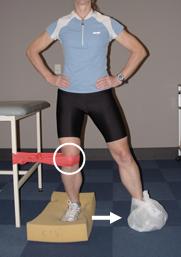 |
| Concentrate on the alignment of your arthritis leg hip, knee and ankle – **position your knee over your foot** against the pull of the Thera-band throughout.  Do 3 sets of 10 repetitions with a break of 30-60 seconds between sets. | |

**Exercise 2: Sideways exercise**

**Level 4:** Sliding with Thera-band and foam and eyes closed

| Starting position:  Place a loop of Thera-Band around your arthritis knee and the leg of a table. This will provide a pull outwards on your knee that you must resist by aligning your knee over your foot through the whole exercise.  Standing on your arthritis leg on your **foam cushion**, with your non-study leg on a sliding surface.Sliding can be achieved by using a towel on smooth flooring or a plastic bag on the foot for carpet.  **Close your eyes.** | 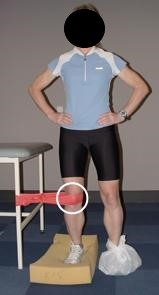  **Arthritis knee** |
| --- | --- |
| Exercise:  **Slowly** slide out sideways with the non-study leg while bending your arthritis knee. Start with sliding just a few inches and progress to larger slides as you gain control.  Keep your weight on your arthritis leg through the whole exercise. | 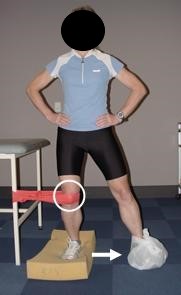 |
| Concentrate on the alignment of your arthritis leg hip, knee and ankle – **position your knee over your foot** against the pull of the Thera-band throughout.  Do 3 sets of 10 repetitions with a break of 30-60 seconds between sets. | |

**Exercise 3: Hip muscle strengthening**

Level 1: Wall push

| Starting position:  Stand sideways to a wall with non-study leg against the wall.  Slightly bend arthritis knee to about 15-20°.  Lift the non-study leg just off the floor so that hip, thigh and knee are touching the wall.  Exercise:  Push your non-study leg into the wall and **hold for 20.** Return you foot to the floor and rest for a few seconds. | 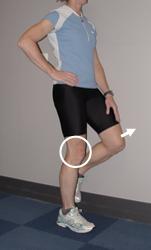  **Arthritis knee** |
| --- | --- |
| Concentrate on the alignment of your arthritis leg hip, knee and ankle – **position your knee over your foot** throughout.  Do 2 sets of 5 repetitions with a break of 30-60 seconds between sets. | |

**Exercise 3: Hip muscle strengthening**

Level 2: Wall push with deeper knee bending

| Starting position:  Stand sideways to a wall with non-study leg against the wall.  Slightly bend arthritis knee to about 15-20°.  Lift the non-study leg just off the floor so that hip, thigh and knee are touching the wall. | 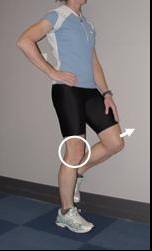  **Arthritis knee** |
| --- | --- |
| Activity:  Push non-study leg into the wall.  While continuing to push into the wall, slowly bend your arthritis knee to a maximum of 45°.  Straighten your knee and return you foot to the floor and rest for a few seconds. | 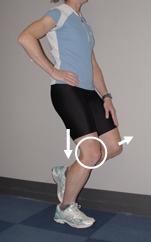 |
| Concentrate on the alignment of your arthritis leg hip, knee and ankle – **position your knee over your foot** throughout.  Do 2 sets of 5 repetitions with a break of 30-60 seconds between sets. | |

**Exercise 3: Hip muscle strengthening**

Level 3: Crab walking + red Thera-band

| Starting position:  Place a loop of red Thera-Band around both ankles so that there is tension when ankles are separated 10cm. Slightly bend both knees.  For safety, you should stand facing a table, a kitchen bench or a wall which you can reach if you lose balance. | 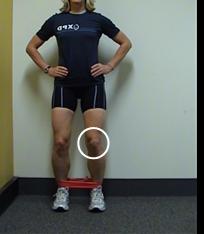  **Arthritis knee** | |
| --- | --- | --- |
| Exercise:  Step sideways against the pull of the Thera-band.  Do not twist or turn your body or legs. Your feet must point forwards while you are stepping sideways. | 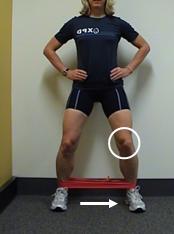 | 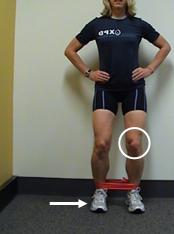 |
| Concentrate on the alignment of both your arthritis and non-study legs – **position your knee over your foot** throughout.  Do a total of **30 steps in each direction**. For example, you can do all 30 in one direction around a table before changing direction. Or you can do 5 in one direction along a kitchen bench, change direction and do 5 back to the start and repeat this 6 times to reach your total of 30 in both directions. | | |

**Exercise 3: Hip muscle strengthening**

Level 4: Crab walking + black Thera-band

| Starting position:  Place a loop of black Thera-Band around both ankles so that there is tension when ankles are separated 10cm. Slightly bend both knees.  For safety, you should stand facing a table, a kitchen bench or a wall which you can reach if you lose balance. | 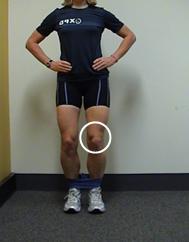 | |
| --- | --- | --- |
| Exercise:  Step sideways against the pull of the Thera-band.  Do not twist or turn your body or legs. Your feet must point forwards while you are stepping sideways. | 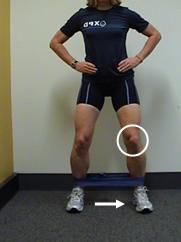 | 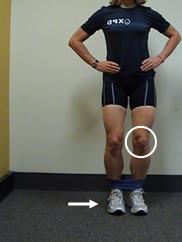 |
| Concentrate on the alignment of both your arthritis and non-study legs – **position your knee over your foot** throughout.  Do a total of **30 steps in each direction**. For example, you can do all 30 in one direction around a table before changing direction. Or you can do 5 in one direction along a kitchen bench, change direction and do 5 back to the start and repeat this 6 times to reach your total of 30 in both directions. | | |

**Exercise 4: Knee muscle strengthening**

Level 1: Wall squats

| Starting position:  Stand with your back to a wall, feet 10cm apart and 15cm away from the wall.  You may like a towel behind your hips to help you slide down the wall. | 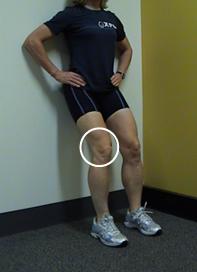  **Arthritis knee** |
| --- | --- |
| Exercise:  Slide **slowly** down the wall until your knees are bent about 30°, then slowly slide up again.  *“Down, 2, 3, hold, 2, 3, up, 2, 3”*  Your knees should stay in line with your feet. | 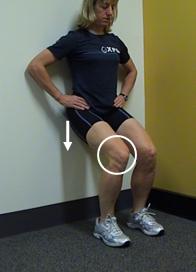 |
| Concentrate on the alignment of both your arthritis and non-study legs – **position your knee over your foot** throughout.  Do 3 sets of 10 repetitions with a break of 30-60 seconds between sets. | |

**Exercise 4: Knee muscle strengthening**

Level 2: Wall squats with more weight on arthritis leg

| Starting position:  Stand with your back to a wall, feet 10cm apart and arthritis leg 15cm away from the wall. Your non-study side can be (a) further forward or (b) level with the arthritis leg but with your body shifted over the arthritis leg. You **must** have **more weight on your arthritis leg through the whole exercise**.  You may like a towel behind your hips to help you slide down the wall. | (a)  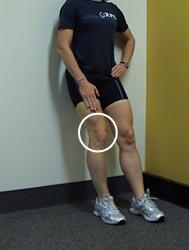  **Arthritis knee** | (b)  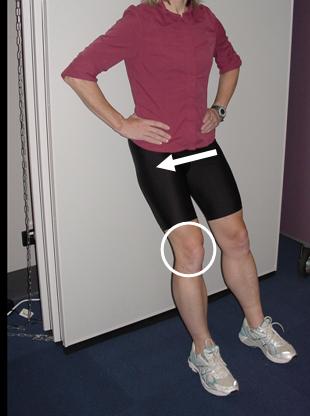  **Arthritis knee** |
| --- | --- | --- |
| Exercises:  Slide **slowly** down the wall until your arthritis knee is bent about 30°, then slowly slide up again.  *“Down, 2, 3, hold, 2, 3, up, 2, 3”*  Your knees should stay in line with your feet.  You may use some scales under arthritis leg to check you keep more weight on that side. | 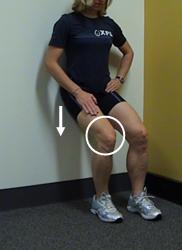 | |
| Concentrate on the alignment of your arthritis leg hip, knee and ankle – **position your knee over your foot** throughout.  Do 3 sets of 10 repetitions with a break of 30-60 seconds between sets. | | |

**Exercise 4: Knee muscle strengthening**

Level 3: Chair stands

| Starting position:  Sit on a standard height (eg. kitchen) chair, with your feet parallel and hip width apart.  Reach your hands out in front. | 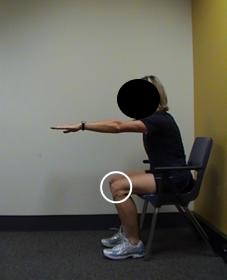  **Arthritis knee** | |
| --- | --- | --- |
| Exercise:  Stand up from the chair slowly (count 4 seconds) without using your hands.  Slowly return to sitting (count 4 seconds).  *“Up, 2, 3, 4, down, 2, 3, 4”* | 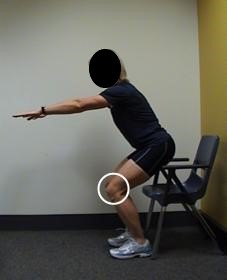 | 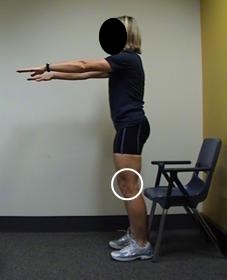 |
| Concentrate on the alignment of both your arthritis leg and your non-study leg – **position your knee over your foot** throughout.  Do 3 sets of 10 repetitions with a break of 30-60 seconds between sets. | | |

**Exercise 4: Knee muscle strengthening**

**Level 4:** Chair stands with more weight on arthritis leg

| Starting position:  Sit on a standard height (eg. kitchen) chair, with your feet hip width apart.  Take **more weight on your arthritis leg** by either (a) placing your non-study leg further forward, or (b) shifting your feet sideways so your arthritis leg is in front of your body.  Reach your hands out in front. | | **(a)**  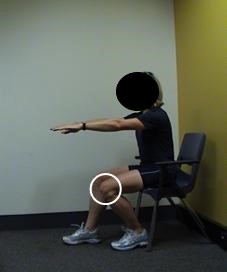  **Arthritis knee** | | **(b)**  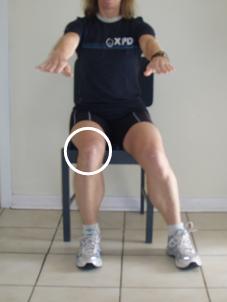  **Arthritis knee** |
| --- | --- | --- | --- | --- |
| Exercise:  Stand up from the chair slowly (count 4 seconds) without using your hands.  Slowly return to sitting (count 4 seconds).  *“Up, 2, 3, 4, down, 2, 3, 4”*  Concentrate on the alignment of your arthritis leg hip, knee and ankle – **position your knee over your foot** throughout.  You **must** have **more weight on your arthritis leg through the whole exercise.** | 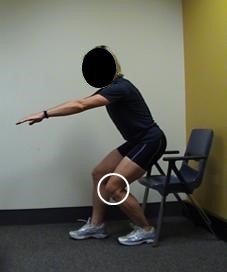  **(a)**  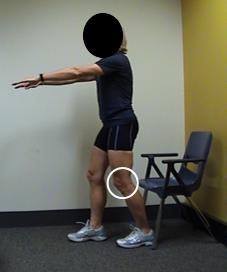  Do 3 sets of 10 repetitions with a break of 30-60 seconds between sets. | | 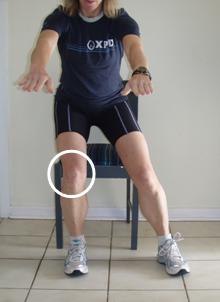  **(b)**  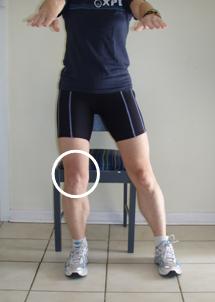 | |
|  | | | | |

**Exercise 5: Step-ups**

**Level 1:** Step-ups

| Starting position:  Place your arthritis leg onto a step in front of you.  Use a hand support (back of chair or handrail) for balance. | 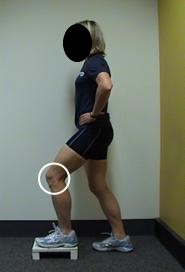  **Arthritis knee** | | |
| --- | --- | --- | --- |
| Exercise:  Step up onto the step slowly, carefully controlling the movement of your arthritis knee.  Just lightly touch your non-study leg to the step, and then step it back down slowly to the start position.  Your weight should be on your arthritis leg through the whole exercise. | 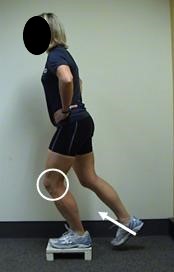 | 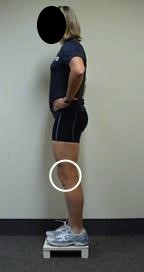 | 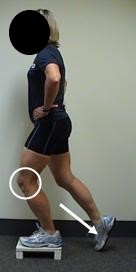 |
| Concentrate on the alignment of your arthritis leg hip, knee and ankle – **position your knee over your foot** throughout.  Do 3 sets of 10 repetitions with a break of 30-60 seconds between sets. | | | |

**Exercise 5: Step-ups**

**Level 2:** Step-ups with weight

| Starting position:  Hold 2kg of weight (a) against your chest, (b) in each hand, (c) in one hand while holding on for balance with the other, or (d) in a backpack. | 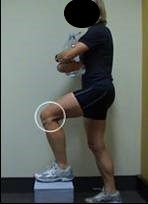  **Arthritis knee**  **(a)** | | 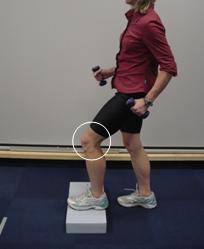  **Arthritis knee**  **(b)** | |
| --- | --- | --- | --- | --- |
| Place your arthritis leg onto a step in front of you.  Weight can be a 2L milk bottle filled (2kg) or half filled (1kg) with water. | | | | |
| Exercise:  Step up onto the step slowly, carefully controlling the movement of your arthritis knee.  Just lightly touch your non-study leg to the step, and then | 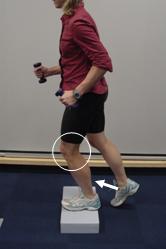 | 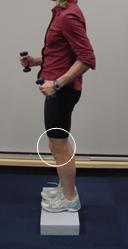 | | 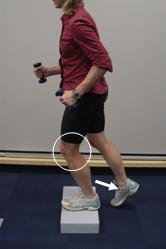 |
| step it back down slowly to the start position.  Your weight should be on your arthritis leg through the whole exercise.  Concentrate on the alignment of your arthritis leg hip, knee and ankle – **position your knee over your foot** throughout.  Do 3 sets of 10 repetitions with a break of 30-60 seconds between sets. | | | | |

**Exercise 5: Step-ups**

**Level 3:** Forward touch downs

| Starting position:  Stand on the step.  Use a hand support (back of chair or handrail) for balance. | 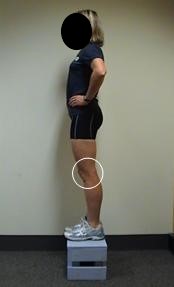  **Arthritis knee** | |
| --- | --- | --- |
| Exercise:  Controlling the movement of your arthritis knee, reach your non-study side towards the floor in front. If you can reach the floor, just touch it lightly. Return to the starting position.  Your weight should be on your arthritis leg through the whole exercise. | 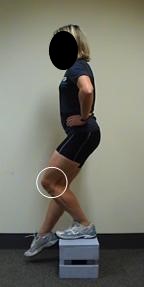 | 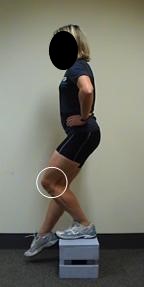 |

| Concentrate on the alignment of your arthritis leg hip, knee and ankle – **position your knee over your foot** throughout.  Do 3 sets of 10 repetitions with a break of 30-60 seconds between sets. |
| --- |

**Exercise 5: Step-ups**

**Level 4:** Forward touch downs with weight

| Starting position:  Stand on the step.  Hold 2kg of weight (a) against your chest, (b) in each hand, (c) in one hand while holding on for balance with the other, or (d) in a backpack.  Weight can be a 2L milk bottle filled (2kg) or half filled (1kg) with water. | 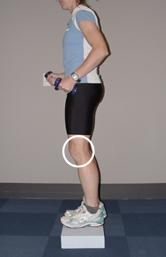  **Arthritis knee**  **(b)** | |
| --- | --- | --- |
| Exercise:  Controlling the movement of your arthritis knee, reach your non-study side towards the floor in front. If you can reach the floor, just touch it lightly. Return to the starting position.  Your weight should be on your arthritis leg through the whole exercise. | 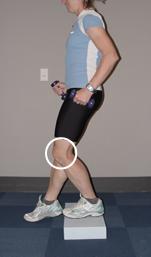 | 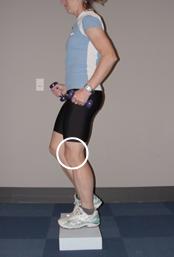 |
| Concentrate on the alignment of your arthritis leg hip, knee and ankle – **position your knee over your foot** throughout.  Do 3 sets of 10 repetitions with a break of 30-60 seconds between sets. | | |

**Appendix 3 – Guide to progression for weight bearing functional exercise program**

| **Level** | **Repetitions** | **Progression** |
| --- | --- | --- |
| **Exercise 1. Forwards/ backwards** | | |
| 1. Sliding | 3 sets of 10 with break of 30-60 seconds between sets | Weeks 1, 2 and 3 |
| 2. Sliding with Thera-band  [Choose a Thera-band colour appropriate for the patient’s ability to ‘medialise’ their knee.] | 3 sets of 10 with break of 30-60 seconds between sets | Weeks 4, 5 and 6 |
| 3. Stepping | 3 sets of 10 with break of 30-60 seconds between sets | Weeks 7, 8 and 9 |
| 4. Stepping with Thera-band  [Choose a Thera-band colour appropriate for the patient’s ability to ‘medialise’ their knee.] | 3 sets of 10 with break of 30-60 seconds between sets | Weeks 10, 11 and 12. |
| **Exercise 2. Sideways exercise** | | |
| 1. Sliding | 3 sets of 10 with break of 30-60 seconds between sets | Weeks 1, 2 and 3 |
| 2. Sliding with Thera-band  [Choose a Thera-band colour appropriate for the patient’s ability to ‘medialise’ their knee.] | 3 sets of 10 with break of 30-60 seconds between sets | Weeks 4, 5 and 6 |
| 3. Sliding with Thera-band and foam [Choose a Thera-band colour appropriate for the patient’s ability to ‘medialise’ their knee.] | 3 sets of 10 with break of 30-60 seconds between sets | Weeks 7, 8 and 9 |
| 4. Sliding with Thera-band, foam and eyes closed [Choose a Thera-band colour appropriate for the patient’s ability to ‘medialise’ their knee.] | 3 sets of 10 with break of 30-60 seconds between sets | Weeks 10, 11 and 12. |
| **Exercise 3. Hip muscle strengthening** | | |
| 1. Wall push | 20 second holds with short break between efforts. Two sets of 5 with break of 30-60 seconds between sets. | Weeks 1, 2 and 3 |
| 2. Wall push with knee bending | Short break between efforts. Two sets of 5 with break of 30-60 seconds between sets. | Weeks 4, 5 and 6 |
| 3. Crab walking with red Thera-band | Total of 30 steps in each direction. [May do all 30 before changing direction or may do in smaller groups depending on available space] | Weeks 7, 8 and 9 |
| 4. Crab walking with black Thera-band | Total of 30 steps in each direction. [May do all 30 before changing direction or may do in smaller groups depending on available space] | Weeks 10, 11 and 12. |
| **Exercise 4. Knee muscle strengthening** | | |
| 1. Wall squats | 3 sets of 10 with break of 30-60 seconds between sets | Weeks 1, 2 and 3 |
| 2. Wall squats with more weight on arthritis leg | 3 sets of 10 with break of 30-60 seconds between sets | Weeks 4, 5 and 6 |
| 3. Chair stands | 3 sets of 10 with break of 30-60 seconds between sets | Weeks 7, 8 and 9 |
| 4. Chair stands with more weight on arthritis leg | 3 sets of 10 with break of 30-60 seconds between sets | Weeks 10, 11 and 12. |
| **Exercise 5. Step-ups** | | |
| 1. Step-ups | 3 sets of 10 with break of 30-60 seconds between sets | Weeks 1, 2 and 3 |
| 2. Step-ups with 2kg weight | 3 sets of 10 with break of 30-60 seconds between sets | Weeks 4, 5 and 6 |
| 3. Forward touch downs | 3 sets of 10 with break of 30-60 seconds between sets | Weeks 7, 8 and 9 |
| 4. Forward touch downs with 2kg weight | 3 sets of 10 with break of 30-60 seconds between sets | Weeks 10, 11 and 12. |
